# Health Star Ratings and Beverage Purchase Intentions: A Study of Australian and New Zealand Hospitality Consumers

**DOI:** 10.3390/foods10112764

**Published:** 2021-11-10

**Authors:** Rob Hallak, Craig Lee, Ilke Onur

**Affiliations:** 1 UniSA Business, City West Campus, University of South Australia, Adelaide, SA 5001, Australia; 2 Department of Tourism, University of Otago, Dunedin 9016, New Zealand; craig.lee@otago.ac.nz; 3 College of Business, Government and Law, Flinders University, Adelaide, SA 5001, Australia; ilke.onur@flinders.edu.au

**Keywords:** healthy beverages, hospitality, consumer behaviour, front-of-package labelling

## Abstract

This study examines the effects of a health star rating system on the attitudes of consumers and their purchase intentions towards beverage products sold in hospitality venues. Previous studies linking health ratings to the food and beverages of consumers mainly focus on fast-moving consumer goods and retail purchasing. However, purchasing patterns in hospitality and foodservice environments are distinct as consumers may be less concerned about health and more interested in the dining experience. Thus, this research focuses on: (1) whether the presence of health star ratings on beverage products influences the willingness of consumers to purchase in the context of the hospitality industry, and (2) identifying the demographic and psychographic factors influencing these behavioural intentions. Using Ordinary Least Squares regression to analyse data from an e-survey of 1021 consumers in Australia and New Zealand, the study found that health star ratings do have an impact on the willingness of consumers to purchase healthy beverages. Specifically, psychographic segmentation around ‘health goals’ is far more pertinent to understanding purchase behaviour in a hospitality setting than age, gender, income, or country. The findings present new insights into the importance of health star labelling on beverages and the purchase intentions of consumers.

## 1. Introduction

Global growth in the foodservices sector has witnessed an increase in the amount of food and beverages purchased from hospitality businesses such as restaurants, take-away, and pubs. In the United Kingdom (UK), 20% of people eat out at least weekly, while in the US more household food expenditure goes to eating out than at home [1,2]. Between 2009 and 2019, food and drink sales in the US foodservice industry increased steadily: from USD 452 billion in 2009 to USD 773 billion in 2019. Despite a decline due to COVID-19 restrictions, foodservice sales of USD 621 billion in 2020 were still higher than most of the preceding 20 years [3]. Similar trends have been observed in the UK, with an increase in restaurant and mobile foodservice turnover from GBP 23 billion in 2010 to GBP 42 billion in 2019 [4].

Dining out accounts for 27% of weekly household food and drink expenditure in Australia, amounting to over AUD 45 billion per year [5,6,7]. The emergence of food delivery apps has also witnessed growth in restaurant and foodservice consumption, with online food delivery platforms (including Uber Eats, Menulog, and Deliveroo) experiencing a 72% revenue increase between 2014 and 2019 [8]. The onset of COVID-19 has also increased the uptake of meal delivery services, with predictions this will continue to rise post-pandemic [9] (p. 27).

This increasing consumption of food away from home highlights the need and opportunity for hospitality/foodservice businesses to innovate and improve the supply of healthier products [10], as well as provide better health information [11,12]. Hospitality businesses generate 40% of their revenues from the sale of beverage products [13]; however, many of these beverages are high in both sugar and calories and are associated with increased risk of Type 2 diabetes and cardiovascular disease [14,15,16]. Consumer demand for healthy and functional beverages has increased in recent years, with sales reaching AUD 2.1 billion in 2020 [17]. These beverages include ready-to-drink teas, superfruit 100% juices such as pomegranate juice, cherry juice, cloudy pear juice, bottled water, herbal teas, and kombucha products [13].

Consumers have developed greater awareness about what constitutes a healthy product and look at front-of-package (FoP) labelling to determine the attributes of a product. FoP nutrition labelling is intended to provide consumers with easy-to-understand information and guidance to inform healthier nutritional choices [18]. There are several approaches to FoP labelling, with evidence suggesting they can affect the perceptions of consumers in positive or negative ways [19]. In principle, nutritional labelling is a powerful communication tool to nudge consumer behaviour, but, it needs to be able to draw the attention of consumers, come from a trustworthy source, be simple to interpret, and enable quick comparisons [19].

A certified Health Star Rating (HSR) system is a nutritional FoP labelling approach introduced to Australia and New Zealand in 2014, where a ‘star rating’ ranges from 0.5 stars (least healthy) to 5 stars (healthiest) [20]. HSRs are shown to have positive outcomes on the food choices of consumers by encouraging selection of healthier products [18,21,22]. In addition, the impact of HSRs on consumer behaviour toward beverages has led to various beverage brands voicing concerns over the move by the Australian government to downgrade the star rating of fruit juices from five stars to two in 2020 [17]. With new categories of purportedly ‘healthy’ beverage products (e.g., ginger shots, protein water) entering the market and expanding distribution in the hospitality industry [13], consumers will seek information on the ‘healthiness’ of these products in making their purchase decisions.

This study expands on the body of work on FoP labelling and consumer behaviour in regard to health product purchasing by focusing specifically on the impacts of a health star rating system on beverage products. In addition, while previous studies have examined beverage-purchasing behaviour in a fast-moving consumer goods (FMCG) context [23] and on university campuses [24], little is known about how consumers make healthy beverage purchase decisions in hospitality and foodservice environments [25]. Even less is known about how different consumer segments respond toward health star ratings on beverages and their impact on intentions to purchase. Thus, the study empirically addresses two overarching research questions:

RQ1. Does the presence of a Health Star Rating on beverages influence hospitality consumers’ willingness to purchase?
RQ2. What demographic and psychographic factors influence these behavioural intentions?

Through this investigation, the research draws on the literature from health, nutrition, hospitality, and consumer behaviour and contributes toward an understanding of consumer responses toward health star ratings on beverage products. It will present implications for beverage manufacturers, hospitality firms, and public health authorities aiming to nudge consumers away from high-calorie sugar-sweetened beverages and support the supply and demand of healthier products through effective communication and labelling. The hospitality and foodservices sector has an important role in public health, and businesses play a part in providing consumers with healthy product options as well as health information to enable consumers to make informed decisions.

Data for this study were collected in 2019 through an e-survey of 1021 consumers in Australia and New Zealand, two countries that are similar in consumer demographics and experiencing a growing demand for healthy food and beverage products [13,17]. Results were analysed through SPSS and STATA using descriptive statistics, factor analysis, and Ordinary Least Squares (OLS) regression.

The rest of the paper is organised as follows. We provide a literature review on FoP nutrition labelling and the HSR system looking at research conducted from a consumer demand perspective. Next, we discuss the research design and data collection before presenting the results of the descriptive statistics and OLS regression. Finally, the discussion and conclusions are presented with implications for research and practice.

## 2. Literature Review 

### 2.1. Health Star Ratings and Consumer Behaviour

An HSR system adopts a largely evaluative approach with a product receiving a ‘star rating’ that can range from 0.5 stars (least healthy) to 5 stars (healthiest) [26]. Scores on the HSR are derived from an algorithm that assesses the food’s nutritional profile [27]. Risky nutritional components (such as energy, sodium, saturated fat, and sugar) are calculated first, and are then modified by healthier components, which include proportions of vegetables, fruits, nuts, fibre, and protein [20]. The subsequent score is then converted to the specific health star rating, with more stars indicating a healthier choice. The popularity of HSRs has witnessed a significant uptake in certain food categories, with evidence suggesting they encourage manufacturers to reformulate their products to attain a higher rating [28].

Public awareness of the HSR has risen since its inception, with 83% of consumers in Australia and 76% in New Zealand being aware when prompted [29,30]. Consumer trust in HSR is also high, with 58% of Australians and 62% of New Zealanders perceiving it to be a credible approach [30]. Recent studies that compared the HSR to other FoP nutrition labelling found the HSR to be the preferred method among consumers across age groups, gender, socioeconomic status, and health status [31]. HSRs have several advantages, such as being simple to use and easy to interpret, plus they can facilitate multiple product comparisons [21,32]. Prior to the implementation of the HSR, food labels in Australia and New Zealand were ineffective in providing consumers with simple, consistent, and accessible information on the nutritional qualities of food and beverage products, especially for consumers from diverse cultural and linguistic backgrounds [28].

Despite consumer awareness and familiarity with HSRs, research on the effectiveness of this rating in determining consumer purchase behaviours remains inconclusive for several product categories [33,34]. For example, the HSR Five-Year Review found that 64% of Australian consumers reported that HSRs influenced their purchase product evaluations; however, only 34% who purchased products with an HSR used it specifically as a tool to select healthier options [30]. A separate longitudinal study also found the HSR system did not significantly affect Australian or New Zealand consumer choice towards healthier options [35]. A recent study by Gorski Findling et al. compared five different FoP nutrition labels. Their survey of 1247 participants found that FoP assisted participants in more accurately gauging the nutritional information of products compared to no label; however, this was not associated with changes in purchase intent [36]. Moreover, the authors argue that a ‘traffic light’ system for FoP labelling may be more effective for consumers in comparing nutrient levels among similar products [36]. Neal et al. suggest that explicit warning labels may be more effective in eliciting healthier choices for packaged food [37]. Similar results were reported in a study of sugary drink consumption, where no significant relationships between product labelling and purchase intention could be determined [38].

However, while some previous studies scrutinise the accuracy and effectiveness of HSRs, there is evidence to suggest that the presence of an HSR on FoP labelling can influence product desirability and purchasing behaviour, irrespective of the actual star rating [35,39]. For example, the Health Star Rating Advisory Committee reported that three in five consumers who reported purchasing a product with an HSR reported that the rating had influenced their purchase decision, with half of those who had been influenced purchasing different products because of the rating [40] (p. 12).

Clearly, gaps remain in our understanding of how HSRs impact consumer purchase intentions especially towards new and supposedly ‘healthy’ beverage products. This study will present new empirical insights on the perceived effectiveness of an HSR in influencing the willingness of consumers to purchase healthy beverages in a hospitality/foodservice environment. This research also addresses the gaps with regards to understanding how different consumer groups, based on demographic and psychographic characteristics, respond differently to HSR. The next section of the literature focuses specifically on HSRs and beverage products.

### 2.2. HSRs and Beverages

A 2019 study analysed the labels of 762 ready-to-drink (non-alcoholic and non-dairy) beverages sold in supermarkets in Australia, measuring the presence of HSR icons (including Energy-Icon which is an optional HSR for beverages) [41]. The analysis revealed that only 6.8% of beverages displayed the Heath Stars, whereas 28.5% presented the Energy-Icon displaying the number of kilojoules and % of Daily Intake. For products that did display an HSR, almost all reported ‘5 stars’ and these were predominantly 100% juice beverages. The researchers conclude that the Energy-Icon with Kilojoule (Kj) and Daily Intake (DI) information should be removed from FoP labelling, recommending instead the mandatory display of Health Stars on beverage products [41]. The extent to which the HSR accurately represents the ‘healthiness’ of products was recently reviewed by the Australia and New Zealand Ministerial Forum on Food Regulation to ensure that beverages high in sugar (such as certain fruit juices) do not receive a 4.5- or 5-star rating, but rather a maximum rating of 4 stars [42,43].

A limitation of previous studies on HSRs is that they take place in the context of FMCG and retail purchasing (i.e., supermarket product purchasing) [30]. The hospitality and foodservice sector plays an important role in public health and nutrition; thus, there is a need to provide consumers with better nutritional information in regard to beverage products, especially considering the wide-spread availability of high-calorie sugar-sweetened beverages [44]. There is also growing concern about artificially sweetened beverages, as these have been associated with higher risk of stroke and dementia [45]. 

A recent US study found that the nutritional quality of food consumed in food service and hospitality venues remains poor [46]. Consumer purchasing behaviour in a hospitality and foodservice environment is distinct when compared to retail purchases. For example, consumers may be more careful about what they eat at home (and in their retail purchases), but when dining out they may be less concerned about health and more interested in the experience [47]. This effect may be further amplified by the social influences on eating, particularly when dining out. In this environment, certain norms (such as not over-indulging, or trying to choose the healthy option) may be relaxed, resulting in increased consumption of unhealthy foods [48,49]. The decisions of hospitality consumers to purchase particular foods or drinks are influenced by a broad range of factors including attitudes, taste and preferences, price, and willingness to pay [50]. Predictably, price remains a key factor in consumer purchasing intent towards healthy beverages. In general, affordability has been proposed as a barrier to purchasing healthy beverage options, especially when compared to the relatively lower prices of less healthy beverages [51]. Health beliefs and health consciousness are also consistent predictors of purchase intent towards healthy food and beverages [11,52]. Consumers who actively pursue a healthy lifestyle are more motivated to make informed and healthy dietary choices [53]. Consumers with higher levels of health consciousness are also more likely to read and assess the descriptive nutritional information on the back of product labels [54,55]. In addition to health beliefs and orientation, evidence suggests women rate higher than men in their preferences towards healthy products [56]. Women are also more likely to be regular purchasers of healthy foods and drinks [57,58] and have a greater inclination to use nutritional labelling for purchase decision making [59,60].

The review of the extant literature has identified the divergence of findings in regard to HSRs and consumer behaviour, highlighting the need to further explore the response of consumers regarding beverage consumption in a hospitality context. Clearly, there are gaps in our understanding of how FoP, specifically an HSR, on beverage products sold in hospitality and foodservice venues affect consumers purchase intentions. Will consumers respond differently to beverage products that present Health Start Ratings? Could an HSR nudge consumers away from purchasing high-calorie sugar-sweetened beverages when dining out and create demand for healthier options? A recent study on healthy beverage in the hospitality industry found that business operators were willing to offer more healthy beverages if there was a clear demand [10]. Moreover, consumer response to HSRs cannot be seen as homogeneous as there are diverse demographic and psychographic factors that affect the perception of HSRs and intentions to purchase health beverages. These factors include attitudes to health, frequency of purchasing, and average amount spent on a purchase, as well as age, gender, and education. These are examined in the present study.

## 3. Materials and Methods

### 3.1. Sample and Data Collection

This research is derived from a larger study on consumer attitudes and purchase behaviours towards healthy beverages sold in hospitality and foodservice venues. Data were collected in 2019 through an electronic survey of 1021 consumers across Australia (AUS) and New Zealand (NZ). Ethics for this study was assessed and approved by the Business School Ethics Committee at the University of South Australia. The sample for this research was drawn from consumer panel data from each country, representative by age, gender, and region. Participants who purchase/eat out at cafes, restaurants, pubs, and takeaways were included in the sample frame. The two countries were chosen due to the significant growth in the hospitality sector and growing demand for healthy food and beverage products [13,17].

### 3.2. Research Instrument and Variables

An e-questionnaire was developed based on common themes from the hospitality management and food research literature [10,61,62]. The term ‘healthy’ was not defined or made explicit to respondents in order “to learn without the complications of definitions what current diners wanted and what their barriers were” [63]. Thus, respondents were asked to self-report the beverages from the menu of a hospitality business they consider as being ‘healthy’ and ‘why’?

Participants were asked to respond to the statement “I would purchase healthy drinks if the product had a health star rating on its label” (1 = Strongly Disagree and 7 = Strongly Agree). This observed criterion variable ‘willingness to purchase if it has HSR’, captures the extent to which consumers are influenced by the presence of an HSR in their willingness to purchase healthy beverage products. The predictor variables for this study include the psychographic characteristics of consumers, specifically regarding attitudes and goals towards health. This is operationalised as ‘healthy eating goals’ and adapted from validated scales from McCarthy et al. [61] and Steptoe et al. [64]. ‘Healthy eating goals’ captures the motivations of individuals to choose healthy food and drink products and maintain a healthy lifestyle, measured by three items: “It’s important to me that the food I eat on a typical day contains vitamins and minerals”; “It’s important to me that the food I eat on a typical day is good for my appearance”; and “It’s important to me that the food I eat on a typical day is nutritious” (1 = Strongly disagree, 7 = Strongly Agree) [61,64]. In addition, we also include several sociodemographic variables for the regression analysis including age, country (AUS or NZ), education, gender, employment status, income, spending when dining out, and the frequency of eating out.

### 3.3. Model Description and Analysis Techniques

Responses from the e-survey were analysed through SPSS and STATA. The criterion variable (‘willingness to purchase if it has HSR’) is a single-item seven-point Likert scale ranging from Strongly Disagree (1) to Strongly Agree (7). Descriptive analysis, factor analysis, and Ordinary Least Squares (OLS) regression are used.

The data analysis was conducted in two sequential steps. First, Principal Component Analysis (PCA) with oblique (Oblimin) rotation was conducted on the three-item Healthy Eating Goals scale. This was to support the reliability and validity of the multi-item scale, enabling it to be used as a predictor variable in the Ordinary Least Squares (OLS) regression. Second, OLS regression was used to examine the effects of the explanatory and control variables on the willingness to purchase healthy drinks if they had an HSR. Demographic variables such as age, education, gender, country, employment, and income are utilised as controls, while frequency of eating out, amount spent while eating out, and healthy eating goals are used as explanatory variables. The OLS regression enables us to capture the variation observed in the dependent variable and identify the explanatory variables contributing to this variation. While using OLS, we treat our dependent variable as a continuous one and the regression results allow us to identify the independent variables with the strongest effect on the willingness of respondents to purchase if an item has HSR.

The regression model is examined as:

(1) $$HSR=\beta_{1}X_{1}+\beta_{2}X_{2}+\beta_{3}X_{3}+u$$

*HSR* is the dependent variable and *X*_1_ is a vector of sociodemographic characteristics of the respondents such as age, gender, education, etc. *X*_2_ represents the two eating out variables: frequency of eating out and the amount spent when eating out. *X*_3_ introduces the psychographic ‘healthy eating goals’ variable. Lastly, ‘u’ signifies the error term, which we assume to be standard normally distributed. We are interested in the marginal effects of the independent variable on HSR, and these marginal effects are directly given by estimates of the coefficients: *β*_1_, *β*_2_ and *β*_3_ and more specifically.

The analysis involves three separate models. Model 1 includes only the *X*_1_ variables, Model 2 introduces variables in *X*_2_, and Model 3 includes all variables. This type of analysis enables us to see how the explanatory and control variables change as new ones are added to the regression. It also enables us to examine if multicollinearity is present.

## 4. Results

### 4.1. Descriptive Analysis

Data were collected from 1021 consumers across AUS (N = 808, 79.14%) and NZ (N = 213, 20.86%) (Appendix A). Results of the 1021 responses (79% AUS, 21% NZ) show an even distribution of male and female respondents, and a relatively even spread of respondents across the six age categories measured. Most respondents were in full- or part-time work, with 24% earning AUD/NZD $40,000–$60,000. Approximately 64% of respondents had tertiary qualifications (including diploma, degree, or postgraduate) (Appendix A). To assess the representativeness of our sample, we compared the age and gender distribution to the census data of their respective country using chi-square difference tests. These tests produced non-significant results indicating that the gender and age distributions of our sample to be similar to the general populations of AUS and NZ (Appendix B).

In terms of purchasing behaviour, over 66% of respondents purchased from hospitality businesses at least once a week, with 20% purchasing three or more times per week. Almost 79% of respondents spent around AUD/NZD $35 or less on each purchase. These results are consistent with data indicating meals away from home accounting for 27% of weekly household expenditure on food and beverages in Australia [5,6]. Analysis of the data also revealed that 70.2% of respondents would purchase healthy drinks if the beverage has ‘proven health benefits’. For the dependent variable (‘willingness to purchase if it has an HSR’), results from 1021 responses reveals 54.9% Agree (and Strongly Agree) to this statement (Mean 4.6, SD 1.63). The following sections present the factor analysis, followed by results of Ordinary Least Squares (OLS) regression models.

### 4.2. Factor Analysis—Healthy Eating Goals

The Principal Component Analysis (PCA) results support a single factor solution (eigenvalue > 1). The component matrix shows each observed item loads highly onto its corresponding factor (>0.6), and scale reliability is also supported with Cronbach’s α > 0.7 (Table 1). The factor analysis results support the validity and reliability (internal consistency) of the scale; thus, we proceed with this construct as a predictor variable in the OLS regression model.

### 4.3. Ordinary Least Squares (OLS) Regression

Model 1 includes only the control variables and the results show a positive association with higher education and our dependent variable, but only for respondents with a Bachelor’s degree or higher (β = 0.473). Compared to those who did not complete high school, respondents with university education care more about the health rating information, and this is reflected with a 0.473 unit increase on the HSR seven-point Likert scale, keeping everything else constant.

In Model 2, we add the purchasing behaviour variables (both the frequency and the amount spent) in addition to the controls used in Model 1. Compared to those who spend AUD/NZD $20 or less in their purchase, consumers spending AUD/NZD $36 or more were significantly less influenced by the presence of an HSR, represented with a 0.35 unit decrease on the HSR scale. This corresponds to more than one-fifth of the standard deviation of our dependent variable. When examining the frequency of eating out, we see that purchasing ‘often’ (3 or more times per week) is significant and positively related to the dependent variable (‘willingness to purchase a healthy beverage if it has an HSR’). Consumers who frequently eat out/purchase meals away from home are more receptive to HSRs and, on average, agree more by 0.382 units for the scale measure of our dependent variable, when compared to those who seldom eat out.

Model 3 builds upon Model 2 by including the Healthy Eating Goals of the respondents. Compared to previous models, we observe a significant change in the age variables. Using 24 years old or younger as the baseline, the regression results show that older age groups (45–54, and 55–64) are negatively related to a willingness to purchase healthy beverages with an HSR, suggesting HSRs are of lesser importance in their beverage purchase decisions. Model 3 also shows that the motivational variable of Healthy Eating Goals has a positive and significant effect on the dependent variable, the largest among all our variables (β = 0.635). The Healthy Eating Goals of consumers have a significant and positive effect on their ‘willingness to purchase a healthy beverage if it has a HSR’. This is reflected by a 0.635 unit increase on the HSR seven-point Likert scale for a one-unit increase in the Healthy Eating Goals measure. In other terms, our estimation predicts a score of 2.5 out of 7 for someone with the lowest healthy eating goals measure, compared to a score of 6 out of 7 for someone with the highest one. Thus, health-conscience consumers are the most receptive towards HSRs in their purchase-making decisions (Table 2).

## 5. Discussion

As an evaluative FoP labelling approach, HSR systems are generally regarded as an effective means of increasing consumer knowledge about the healthiness of food and beverages. However, empirical evidence supporting the efficacy of HSRs on the attitudes and purchase intentions of consumers towards different product categories remains uncertain. It is also unclear how different consumers with varying demographic and psychographic characteristics respond differently to HSRs and their effects on purchase decisions. This study addresses these gaps and contributes to the body of knowledge on consumer perceptions toward HSRs and their influences on purchase intentions, specifically in the context of beverage products within hospitality and foodservice environments. Thus, the study addressed two overarching questions: (1) Does the presence of a Health Star Rating on beverages influence the willingness of hospitality consumers to purchase? and (2) What demographic and psychographic factors influence these behavioural intentions?

### 5.1. HSR Effectiveness

Results of the electronic survey conducted in 2019 with over 1000 consumers across Australia and New Zealand revealed that 70.2% of respondents would purchase a healthy drink if the beverage has proven health benefits. In addition, 55% of respondents Agree (and Strongly Agree) with the statement “I would purchase healthy drinks if the product had a health star rating on its label” (M = 4.6, SD 1.63). These findings contribute to the body of knowledge on the effectiveness of HSRs [33,34,35,39], providing empirical evidence that an HSR system on beverage products can drive the willingness of consumers to purchase. An HSR is perceived as a non-marketer source of information that validates the purported ‘healthiness’ of a beverage product. An HSR system also has the attributes of effective FoP labelling including (1) be able to draw the attention of consumers, (2) come from a trustworthy source, (3) be simple to interpret, and (4) enable quick comparisons [19].

### 5.2. HSR and Consumer Characteristics

The study also examined the extent to which demographic and psychographic factors influence the response of consumers to HSRs. OLS regression identified that education, age, frequency of eating out, amount spent on a purchase, and healthy eating goals are significantly related to the dependent variable. Specifically, education is positively related to HSR perceptions, with bachelors/postgraduate-level education having significant effects. These results parallel findings from the nutrition and health literature that report individuals with higher educational attainment are more interested in the nutritional aspects and ingredients of foods [65].

Results also suggest age to be a determining factor, with older age groups (45–64) being significantly and negatively related to the criterion. These findings are unexpected, in contrast to previous studies reporting older adults to be more likely to choose healthy products [50,57,66]. However, much of the existing research has investigated retail purchasing behaviour, which is distinct from hospitality and foodservice purchasing behaviours. Indeed, there is evidence to suggest that older adults in particular are more likely to dine out for the socialisation opportunities rather than for nutritional considerations or convenience [67,68]. When dining out, the overall energy intake of older adults also tends to be significantly higher [69,70], possibly as the activity is viewed as a ‘treat’ or an experience to be enjoyed [71]. These factors may account for the discrepancies between retail purchasing behaviour and hospitality purchasing behaviour as observed in our data.

Our data found that frequently dining out (three or more times a week) has a significant positive effect on willingness to purchase beverages that display HSRs. The analysis also found a negative relationship between increase in spending and the dependent variable, suggesting those who not only purchase frequently but also spend less on their purchases, are more receptive to HSRs. Previous studies suggest consumers may watch what they eat at home but are less careful when dining out [47] due to the novelty/reward factor of eating out. However, our results suggest that for those consumers who dine out often, the line between dining ‘out’ and ‘in’ may be blurred, resulting in more health-conscious purchase intentions away from home. Thus, consumers who eat out often (but spend less on a purchase) are more receptive to purchasing beverages with an HSR.

### 5.3. Healthy Eating Goals and Response to HSR

The results found no significant relationships between gender, country (AUS or NZ), or household income on the HSR dependent variable. However, the motivations of consumers towards healthy eating have a strong significant impact. Healthy Eating Goals was the highest predictor of the criterion, highlighting that psychographic (more than demographic) variables play an important role in consumer responsiveness to HSRs and their willingness to purchase beverage products with HSRs. This is consistent with previous studies finding consumers who actively pursue a healthy lifestyle make more informed and healthier dietary choices [53]. Moreover, consumers interested in health and wellbeing are also more likely to make conscious dietary decisions, such as opting for low-fat food or low-sugar drinks [72,73].

## 6. Conclusions

In conclusion, this study expands on the body of knowledge on FoP labelling and HSRs by examining the extent to which HSRs influence the purchase intentions of consumers of beverages in a hospitality context. We find that HSRs do affect willingness to purchase healthy beverages as they provide authentication of the ‘healthiness’ of a product. Our findings also suggest the presence of heterogeneity among consumer groups, specifically regarding their healthy eating goals affecting consumer responses towards HSRs. In segmenting the market, psychographic variables around ‘health goals’ are far more pertinent in understanding behaviour than demographic variables such as age, gender, income, or country of origin.

These empirical findings present insights into health promotion and nutrition labelling and also have several practical and policy implications. Healthfulness and the importance of nutrition are increasingly understood by consumers, with this understanding also expanding into beverage choices [74]. For beverage manufacturers, our research provides further evidence that HSRs can, and do, drive the willingness of consumers to purchase healthy beverages in a hospitality setting. Thus, the inclusion of HSRs may be considered for all beverages promoted as being healthy, irrespective of market sector.

For hospitality firms, our research suggests that psychographic factors—such as attitudes towards health and healthy eating—are key elements that influence purchasing intentions of healthy beverages. Therefore, developing an understanding of healthy beverage selection among these specific consumer groups will aid the hospitality and foodservice sectors in providing a suitable and appropriately targeted range of beverages. It will also allow hospitality firms to play a part in the promotion of healthier options to consumers in the interest of public health.

For policy makers, our research adds further support to the use of HSRs as an effective policy tool for health promotion in the hospitality industry. The importance of psychographic factors in healthy beverage purchasing behaviour suggests that HSRs have an important role to play as part of broader public health initiatives that seek to improve overall attitudes towards healthiness.

## 7. Limitations and Future Research

In terms of limitations and associated avenues for future research, data for this study were collected from hospitality industry consumers in Australia and New Zealand. There could be specific economic and environmental factors unique to this industry and these consumers that may differ in other settings. In addition, the study focuses on a specific type of FoP labelling (i.e., HSR), as this rating is applicable in Australia and New Zealand where the study took place. Thus, further studies could examine consumer responses towards different types of nutritional labelling, such as the traffic light food system. This will allow for a comparison of different types of food labelling systems in order to identify which standard may be the most effective in influencing healthy choices.

We are also cognizant that HSRs are not applicable to alcoholic beverages, which are a major beverage category in a hospitality dining environment. Moreover, the participants of this study were asked in general terms whether they would purchase a healthy beverage if it displayed an HSR on its label; however, they were not provided with an example of a healthy beverage. Thus, there is an opportunity for future studies to qualitatively explore the perceptions of consumers of HSRs in a hospitality setting. While research has begun in this area, particularly in relation to the perceptions of younger people about the healthiness of beverages [75,76], further exploration is warranted. For instance, developing an understanding of how individuals with different sociodemographic characteristics perceive HSR labelling could provide valuable additional insights. Moreover, it would be useful to explore the interaction between HSR labelling and specific types of healthy beverages and how this affects consumer beverage choice. This could help identify which mix of products and FoP labelling may be most effective in promoting healthy beverage choices.

## Figures and Tables

**Table 1:** Healthy Eating Goals.

Variable	Item Description	Mean (SD)	Factor 1
Healthy Eating Goals 1	It’s important to me that the food I eat on a typical day contains vitamins and minerals	4.7 (1.539)	0.858
Healthy Eating Goals 2	It’s important to me that the food I eat on a typical day is good for my appearance	4.24 (1.587)	0.757
Healthy Eating Goals 3	It’s important to me that the food I eat on a typical day is nutritious	5.08 (1.399)	0.825
Eigenvalue			2.505
Cronbach’s α			0.833

**Table 2:** OLS Regression Results.

Variable	Model 1	Model 2	Model 3
Country (Base: Australia)			
New Zealand	−0.155	−0.152	−0.0251
	(0.137)	(0.137)	(0.127)
Gender (Base: Male)			
Female	0.0754	0.106	−0.0467
	(0.112)	(0.112)	(0.105)
Age (Base: 24 or younger)			
25 to 34	−0.102	−0.0977	−0.227
	(0.191)	(0.190)	(0.177)
35 to 44	−0.133	−0.0792	−0.216
	(0.189)	(0.189)	(0.176)
45 to 54	−0.292	−0.243	−0.464 *
	(0.193)	(0.194)	(0.180)
55 to 64	−0.371	−0.304	−0.492 **
	(0.198)	(0.200)	(0.186)
65 or older	−0.158	−0.0353	−0.389
	(0.260)	(0.263)	(0.246)
Education status (Base: Did not complete high school)			
-High School	0.191	0.186	−0.0610
	(0.221)	(0.221)	(0.206)
Certificate/Diploma	0.322	0.324	0.135
	(0.217)	(0.216)	(0.201)
Bachelor/Post-graduate	0.473 *	0.431	0.0984
	(0.227)	(0.227)	(0.213)
Employment status (Base: Full-time employed)			
Part-time employed	0.0127	0.0296	0.119
	(0.156)	(0.155)	(0.144)
Retired	−0.358	−0.356	−0.150
	(0.247)	(0.247)	(0.229)
No employment	0.0831	0.104	0.157
	(0.166)	(0.167)	(0.155)
Household income (Base: Less than AUD/NZD $40,000)			
AUD/NZD $40,000–$60,000	0.296	0.301	0.285
	(0.159)	(0.159)	(0.147)
AUD/NZD $61,000–$85,000	0.111	0.115	0.174
	(0.183)	(0.183)	(0.170)
AUD/NZD $86,000–$100,000	0.0656	0.0955	0.0545
	(0.201)	(0.201)	(0.187)
AUD/NZD $100,000 or more	0.172	0.181	0.130
	(0.182)	(0.184)	(0.171)
Spending when eating out (Base: AUD/NZD $20 or less)			
AUD/NZD $21–$35		−0.196	−0.166
		(0.125)	(0.116)
AUD/NZD $36 or more		−0.350 *	−0.368 **
		(0.145)	(0.135)
Frequency of eating out (Base: Seldom)			
Often		0.382 *	0.282
		(0.166)	(0.154)
Normal		0.192	0.136
		(0.130)	(0.120)
Healthy Eating Goals			0.635 **
			(0.0527)
Constant	4.359 **	4.265 **	4.703 **
	(0.278)	(0.296)	(0.277)
N	905	905	905
Model R-squared	0.03	0.044	0.18

* significant at 0.05; ** significant at 0.01.

## Data Availability

The data presented in this study are available on request from the corresponding author. The data are not publicly available due to privacy guidelines as stipulated by the funding authority.

## Appendix A

**Table A1:** Summary statistics of the sample.

Variable	N	%
*Country*		
Australia	808	79.14
New Zealand	213	20.86
*Gender*		
Female	508	50
Male	508	50
*Age*		
24 and younger	188	18.41
25–34	181	17.73
35–44	173	16.94
45–54	164	16.06
55–64	151	14.79
65 and over	164	16.06
*Employment Status*		
Employed full-time	311	30.46
Employed part-time	221	21.65
Unemployed	67	6.56
Student	68	6.66
Retired	162	15.87
Homemaker	80	7.84
Self-employed	66	6.46
Unable to work	46	4.51
*Annual Household Income*		
Less than AUD/NZD $40,000	233	25.27
AUD/NZD $40,000–$60,0000	223	24.19
AUD/NZD $61,000–$85,000	151	16.38
AUD/NZD $86,000–$100,000	121	13.12
Over AUD/NZD $100,000	194	21.04
*Education*		
Did not complete high school	89	8.85
Completed high school	270	26.84
Certificate/Diploma	324	32.21
Bachelor degree	228	22.33
Post-graduate degree	95	9.30
Other	15	1.47
*Frequency of Eating Out*		
More than 5 times a week	48	4.70
3–5 times a week	155	15.18
Once or twice a week	474	46.43
Once a month	195	19.10
Only on special occasions	149	14.59
*Spending when Eating Out*		
Less than AUD/NZD $10	61	5.97
AUD/NZD $10–$20	416	40.74
AUD/NZD $21–$35	324	31.73
AUD/NZD $36–$50	144	14.10
More than AUD/NZD $50	76	7.44

## Appendix B

**Table A2:** Chi-square difference tests for age and gender distributions.

Country	Variable	Category	Observed N	Expected N ^a^	Chi-Square	Sig.
Australia	Gender	Male	398	398.9	0.003	0.998
		Female	406	405.2		
	Age	18–24	131	100.9	10.73	0.097
		25–34	147	160.3		
		35–44	137	142.4		
		45–54	129	135.5		
		55–64	120	123.7		
		65+	134	135.4		
New Zealand	Gender	Male	110	104.6	0.545	0.762
		Female	102	107.4		
	Age	18–24	37	24.3	9.561	0.144
		25–34	34	36.4		
		35–44	36	35.3		
		45–54	35	34.2		
		55–64	31	31.2		
		65+	30	39.6		

^a^ Expected N calculated based on Australia and NZ census data.
